# Mitochondrial transplantation therapy inhibit carbon tetrachloride‐induced liver injury through scavenging free radicals and protecting hepatocytes

**DOI:** 10.1002/btm2.10209

**Published:** 2020-12-30

**Authors:** Zizhen Zhao, Yixue Hou, Wei Zhou, Rajendiran Keerthiga, Ailing Fu

**Affiliations:** ^1^ College of Pharmaceutical Sciences, Southwest University Chongqing China

**Keywords:** energy supply, free radical, mitochondrial therapy, UPR^mt^

## Abstract

Carbon tetrachloride (CCl_4_)‐induced liver injury is predominantly caused by free radicals, in which mitochondrial function of hepatocytes is impaired, accompanying with the production of ROS and decreased ATP energy supply in animals intoxicated with CCl_4_. Here we explored a novel therapeutic approach, mitochondrial transplantation therapy, for treating the liver injury. The results showed that mitochondria entered hepatocytes through macropinocytosis pathway, and thereby cell viability was recovered in a concentration‐dependent manner. Mitochondrial therapy could increase ATP supply and reduce free radical damage. In liver injury model of mice, mitochondrial therapy significantly improved liver function and prevented tissue fibrogenesis. Transcriptomic data revealed that mitochondrial unfold protein response (UPR^mt^), a protective transcriptional response of mitochondria‐to‐nuclear retrograde signaling, would be triggered after mitochondrial administration. Then the anti‐oxidant genes were up‐regulated to scavenge free radicals. The mitochondrial function was rehabilitated through the transcriptional activation of respiratory chain enzyme and mitophage‐associated genes. The protective response re‐balanced the cellular homeostasis, and eventually enhanced stress resistance that is linked to cell survival. The efficacy of mitochondrial transplantation therapy in the animals would suggest a novel approach for treating liver injury caused by toxins.

## 1 INTRODUCTION

Toxic chemical intoxication is a significant cause of liver injury and is most common in animals that are exposed to chemical poisoning.^1^ The toxic chemicals, such as carbon tetrachloride (CCl_4_), chloroform, arsenic, and so forth, with short‐term, long term or repeated exposure, can damage liver function, producing acute or chronic hepatitis and developing fibrosis and cirrhosis. Among these chemicals, CCl_4_ is extensively utilized and caused animal liver injury.^2^, ^3^ The primary mechanism of CCl_4_‐induced liver injury is to induce free radical production and damage hepatic mitochondrial function, which represents both reduced mitochondrial mass and impaired metabolism of the remaining mitochondria.^4^ Therefore, the protection of mitochondrial function and prevention of the impairment caused by free radicals have become potential therapeutic approaches in rescuing cell function.^5^, ^6^

It is well known that mitochondria play crucial roles in bioenergy production and survival of cells. Recovery of mitochondrial function is suggested to restore cell viability, which has become a promising strategy for treating mitochondrial dysfunction related diseases.^7^, ^8^ Recently, increasing evidence shows that mitochondria can directly enter cells for medical applications.^9^, ^10^ The purpose of the mitochondrial transplantation therapy is to substitute the intracellular dysfunctional mitochondria with healthy exogenous mitochondria to cure mitochondria‐associated diseases, which will benefit the treatment of the diseases caused by irreversible mitochondrial impairment. For examples, Cowan et al. reported intracoronary exogenous mitochondrial delivery into the ischemic heart for cardioprotection,^11^ and Gollihue et al. stated that healthy exogenous mitochondria could produce functional neuroprotection in the experimental spinal cord damage.^12^ In our recent studies, systematic administration of isolated mitochondria retards liver damage initiated by acetaminophen and a high‐fat diet.^13^, ^14^ The biochemical mechanism of the mitochondria was proved to promote ATP production, as well as powerfully eliminate free radicals. Thus, we assumed that the mitochondrial therapy might recover hepatocytes from free radicals in CCl_4_‐induced injury.

In this study, we administrated the isolated mitochondria into the model mice which was intoxicated by CCl_4_, and the therapeutic effect of the mitochondria was evaluated by biochemical measurements and transmission electron microscope (TEM) observation. In order to elucidate the molecular mechanism of mitochondrial therapy, transcriptomics study was executed for the investigation of the differentially expressed genes (DEGs) and associated signaling pathways involving in the recovery of liver function. The research findings will provide a novel insight into the treatment of free radical‐induced tissue injury.

## 2 RESULTS

### 2.1 Mitochondria entered cells through macropinocytosis

**FIGURE 1:**
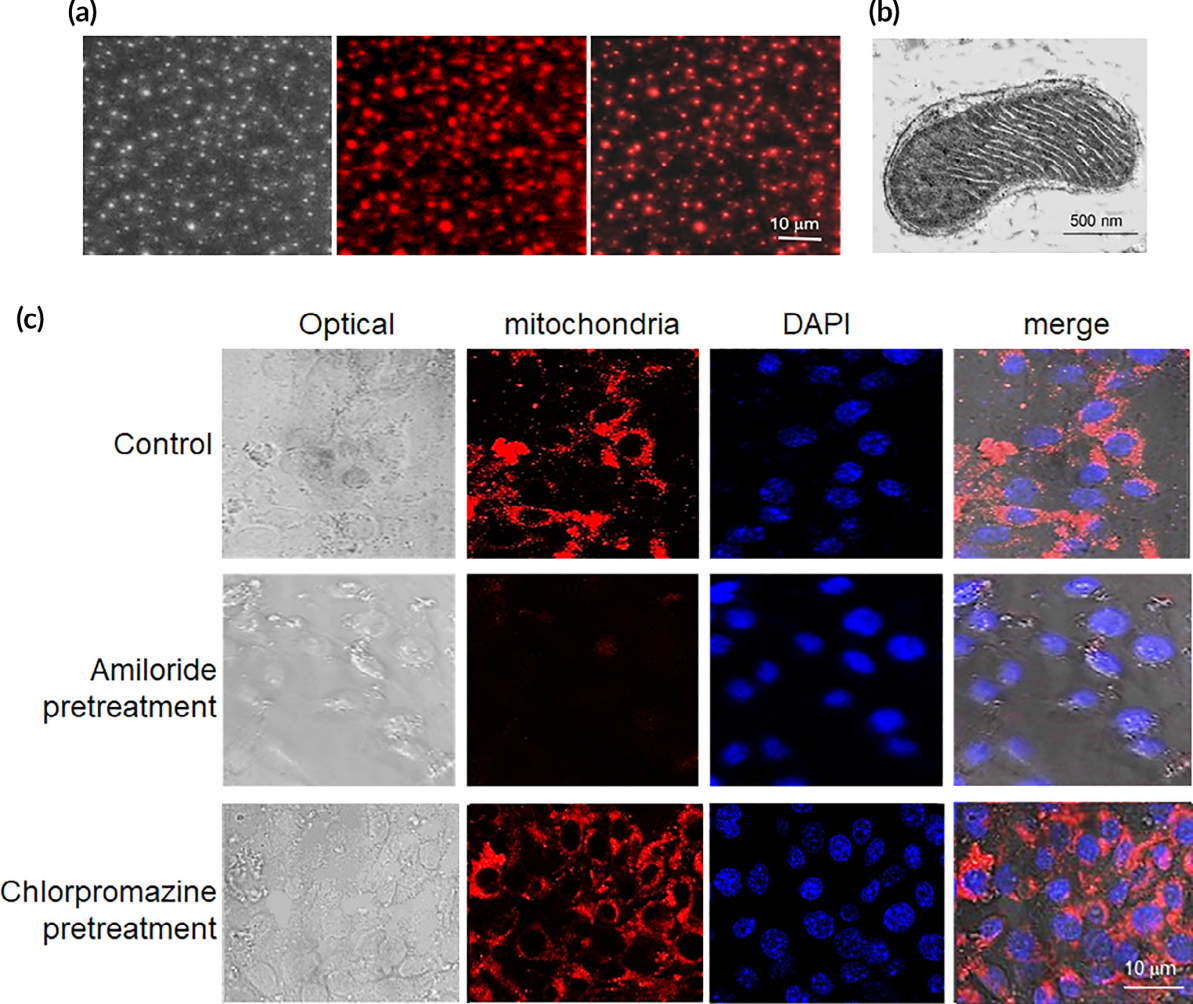
Mitochondria arrived into hepatocyte through macropinocytosis pathway. (a) Isolated mitochondria. Mitochondria were stained by Mitotracker CMXRos, a mitochondrial specific dye. The dye conjugates thiols of mitochondrial proteins with covalent bonding. (b) Mitochondria morphology under TEM. (c) Cell internalization of mitochondria was prevented by macropinocytosis inhibitor. The cells were respectively added 8 × 10^4^ mitochondria, and then were observed under TEM at 4 h after addition

The liver mitochondria showed good dispersion and exhibited red fluorescence observed by confocal microscope (Figure 1a). Mitochondrial bilayer membrane was intact, and cristae was compact and regularly arranged under TEM (Figure 1b).

Endocytosis is a potential energy‐dependent process through which macromolecules enter cells. Here we respectively used inhibitors of clathrin‐mediated endocytosis and macropinocytosis, chlorpromazine and amiloride, to investigate the entry pathway of the mitochondria. After chlorpromazine addition into cell media, fluorescence intensity did not show apparent changes compared with control cells (Figure 1c). However, cells treated with amiloride exhibited very weak fluorescence, indicating that the cell internalization of mitochondria was inhibited. Thus, mitochondria entered cells through macropinocytosis pathway in the study.

### 2.2 Mitochondria prevented CCl_4_‐induced cell injury

**FIGURE 2:**
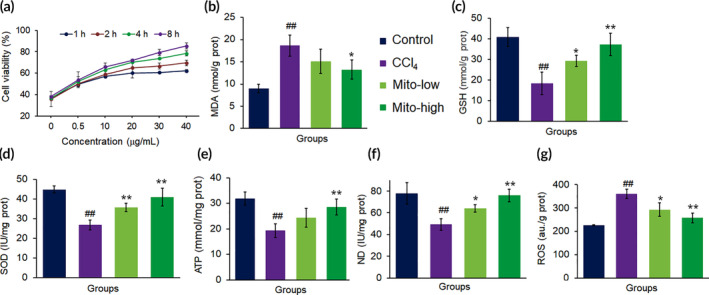
The mitochondria rescued hepatocytes damaged by CCl_4_. (a) Mitochondria increased cell viability in concentration‐ and time‐dependent manners. (b) MDA content decreased, whereas (c) GSH, (d) SOD, (e) ATP, and (f) ND increased in cell homogenates after the mitochondria were introduced into the cell media for incubation 8 h. Mito‐low, low concentration mitochondria (20 μg/ml); Mito‐high, high concentration mitochondria (40 μg/ml). Data were expressed as mean ± SD. ^##^ *p <* 0.01 comparison with the normal control; *p < 0.05, **p < 0.01 compared with the CCl_4_ group. The values were averaged for six independent experiments

CCl_4_ is an industrial solvent with substantial hepatotoxicity. In hepatocytes, CCl_4_ is metabolized to produce trichloromethyl free radical (CCl_3_˙), and further the CCl_3_˙ radical treated with oxygen to produce trichloromethyl peroxy free radical species (CCl_3_OO˙). The two species of free radicals can bind to lipids and proteins of biological membranes, leading to hepatocyte impairment. In this study, CCl_4_ significantly decreased cell viability (Figure 2a), meanwhile the content of MDA (a lipid peroxides product) increased, and the levels of anti‐oxidants (SOD and GSH) and ATP reduced (Figure 2b–e). However, after the addition of exogenous mitochondria into the hepatocytes cell media, cell viability elevated with the concentration‐ and time‐dependent pattern. The levels of anti‐oxidants, ATP and mitochondrial ND, increased, while ROS level significantly reduced after mitochondrial treatment (Figure 2f). The results suggested that mitochondria could increase cell viability through increasing ATP production and diminishing free radical.

### 2.3 Mitochondria distributed in the liver after intravenous injection

**FIGURE 3:**
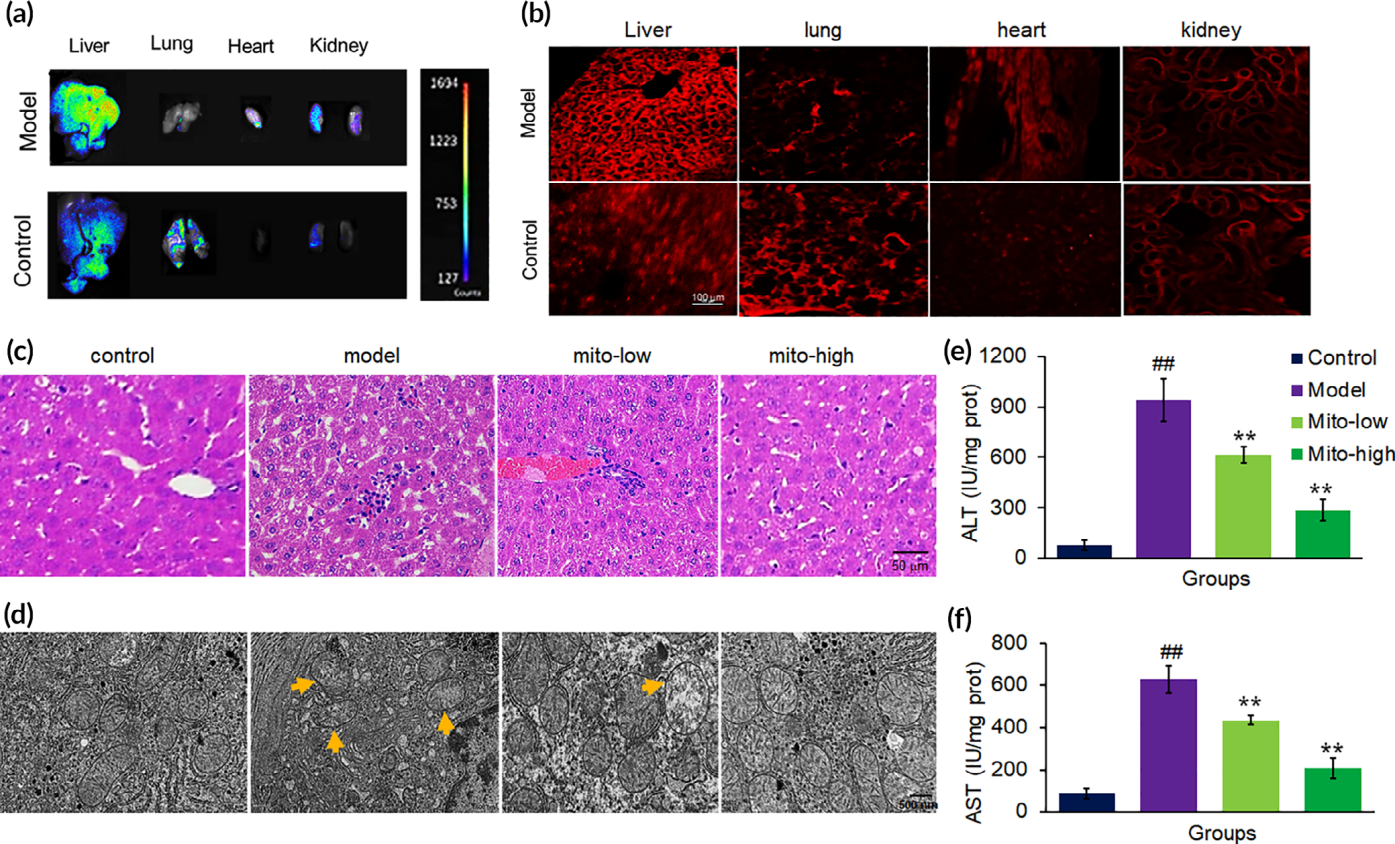
Mitochondrial improved mouse liver function. (a) Tissue imaging with in vivo imaging after 4 h injection of mitochondria (0.4 mg/kg body weight). (b) Tissue sections under confocal microscopy after mitochondrial administration for 4 h. Mitochondria were labeled by Mitotracker CMXRos. (c) Liver sections stained with HE from mice treated with saline, CCl_4_, CCl_4_ + mito‐low (low dosage of mitochondria; 0.2 mg/kg body weight), and CCl_4_ + mito‐high (high dosage of mitochondria; 0.4 mg/kg body weight). (d) Mitochondrial morphology of livers under TEM. (e) Serum ALT activity. (f) Serum AST activity. Data were expressed as mean ± SD (*n* = 10 mice for each group). ^##^ *p* < 0.01 compared with the normal control; **p < 0.01 compared with the CCl_4_ group

After mice were administrated with fluorescence‐labeled exogenous mitochondria (0.4 mg/kg body weight) for about 4 h, tissue fluorescence was observed under in vivo imaging, and fluorescence of the tissue sections was recorded by confocal microscope (Figure 3a,b). The results indicated that the mitochondria through intravenous injection distributed in the liver, lung and kidney, and a small amount in heart. Besides, the livers impaired by CCl_4_ exhibited the strongest fluorescence compared to other tissues and control livers, which might be due to the destruction of the liver structure by CCl_4_, resulting in an increase in the amount of mitochondrial entry.

### 2.4 Mitochondria inhibited CCl_4_‐induced chronic liver injury and fibrosis

CCl_4_ is widely used to establish animal model of liver injury. The pathological process induced by CCl_4_ undergoes acute and chronic injury, subsequently liver fibrosis, and finally cirrhosis. When CCl_4_ dissolved in olive oil is administrated to mice for only once, it can induce acute hepatocellular injury.^15^ When CCl_4_ is repeatedly injected, the impairment becomes aggravating and irreversible, followed by liver fibrosis and cirrhosis.

Here we respectively administrated the mice for 3 and 5 weeks' with CCl_4_ injection to induce chronic liver injury and fibrosis model to examine the effect of mitochondria therapy. As shown in Figure 3c, CCl_4_ caused the marked histopathological changes on HE sections, including hepatocyte necrosis, sinusoidal dilatation, and inflammatory cell infiltration after CCl_4_ injection for 3 weeks. Under TEM, mitochondria were damaged by CCl_4_ administration, evidenced as mitochondrial swelling, broken mitochondrial membrane, irregular crista arrangement, and vacuolar structure (Figure 3d). However, mitochondrial ultrastructure is improved after mitochondrial therapy in CCl_4_‐poisoned mice. In addition, enhanced serum ALT and AST levels in CCl_4_‐treated mice decreased after mitochondrial administration (Figure 3e,f). Thus, the study demonstrated that the therapeutic effect of mitochondria against CCl_4_‐produced free radical damage and cellular degeneration in mouse liver tissue.

**FIGURE 4:**
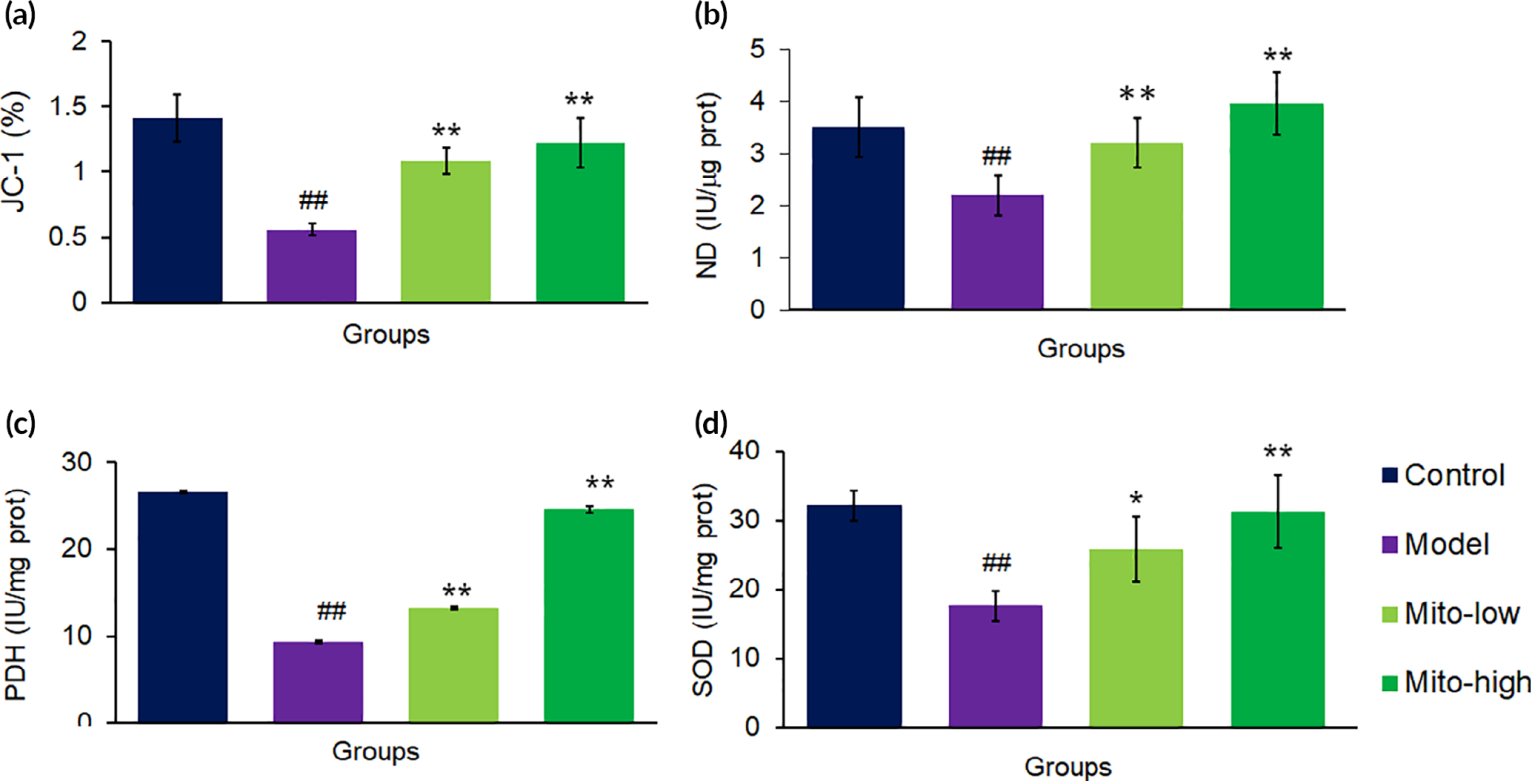
Biochemical measurement of mitochondrial activities in mouse livers after mitochondrial treatment. (a) JC‐1 assay. Also, activities of mitochondrial ND (b) PDH (c) and SDH (d) were respectively measured after mitochondria were administered into the mice. Data were expressed as mean ± SD of the mean (*n* = 10 mice for each group). ^##^ *p* < 0.01 compared with the control; *p < 0.05, **p < 0.01 comparison with the CCl_4_ group

To further determine the mitochondrial function, we measured the levels of mitochondrial membrane potential and respiratory chain‐related enzymes. The results showed that mitochondrial therapy almost wholly reversed the decreased membrane potential caused by CCl_4_ (Figure 4a), and the activities of both SDH and PDH increased (Figure 4c,d).

**FIGURE 5:**
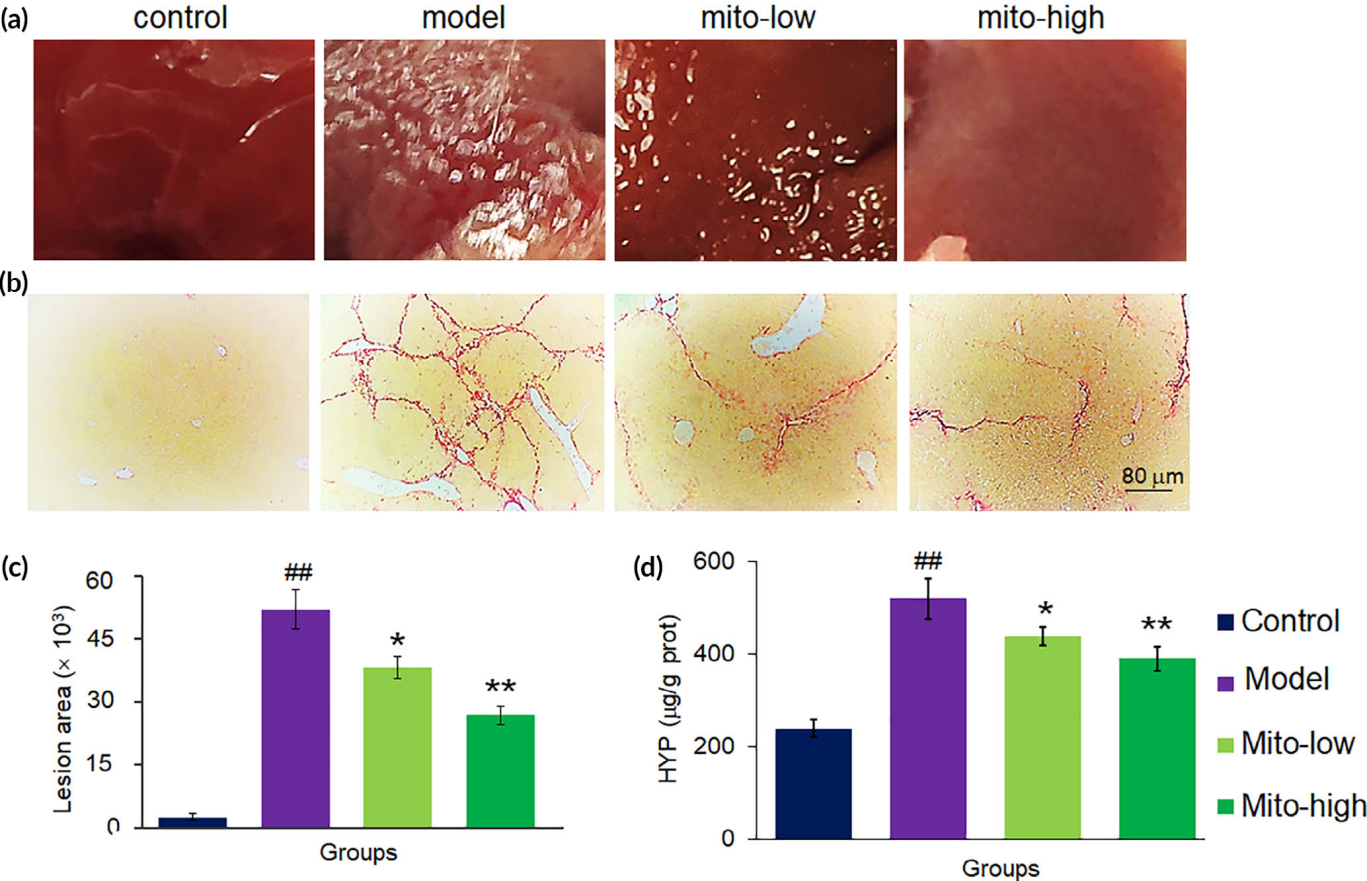
Mitochondria prevented liver fibrosis caused by CCl_4_. (a) Mouse liver surface of each group. (b) Liver sections stained by Sirius red. (c) Quantitative determination of fibrotic area. (d) Hydroxyproline level of liver tissue in each group. Data were expressed as mean ± SD (*n* = 10 mice for each group). ^##^ *p* < 0.01 compared with the control; *p < 0.05, **p < 0.01 compared with the CCl_4_ group

Moreover, CCl_4_‐induced fibrosis is a stable model over a 5‐week period following cessation of CCl_4_ exposure. We utilized this model of chemically produced fibrosis to evaluate the anti‐fibrosis potential of the mitochondria by continuously administering them for 7 days. The healthy liver showed a smooth surface, while it became rough after CCl_4_ intoxication (Figure 5a). Mitochondrial therapy improved the liver surface morphology and shrunk the fibrotic area on sections by Sirius red staining (Figure 5b,c). Besides, a biochemical assay of hydroxyproline content indicated that the hydroxyproline, a biomarker of fibrosis, was suppressed in the liver tissues of the model mice after the treatment with the mitochondria (Figure 5d).

### 2.5 The functional assortment of DEGs

RNA‐seq datasheet showed that DEGs with 2.5 or higher folds between mito‐treated and control groups were differentiated upon the guideline of log_2_(Fold Change) > 1 and the *p*‐value <0.05. The quantity of 713 DEGs were statistically screened out from the constructed transcripts, inclusive of 168 up‐regulated DEGs and 545 down‐regulated DEGs. These set of DEGs were functionally annotated to the reference Genome of *Mus_musculus* (GRCm38.dna.primary_assembly.fa.gz), as well as against public protein database, for example, NOG, UniProtID, and Kyoto Encyclopedia of Genes and Genomes (KEGG). GO enrichment was conducted to determine the DEGs' functional classification. It was shown that the 168 up‐regulated DEGs and the 545 down‐regulated DEGs were further divided into three divisions, inclusive of molecular function, cellular component, and biological process. The molecular function cluster was further divided into 31 subcategories, cellular component into 26 subcategories, and biological process into 17 subcategories. The subcategories of “catalytic activity” and “small molecular binding” are the two most abundant GO terms within the molecular function category, and the subdivisions of “cell cycle process” and “response to stress” were the dominant GO terms in other two clusters.

**FIGURE 6:**
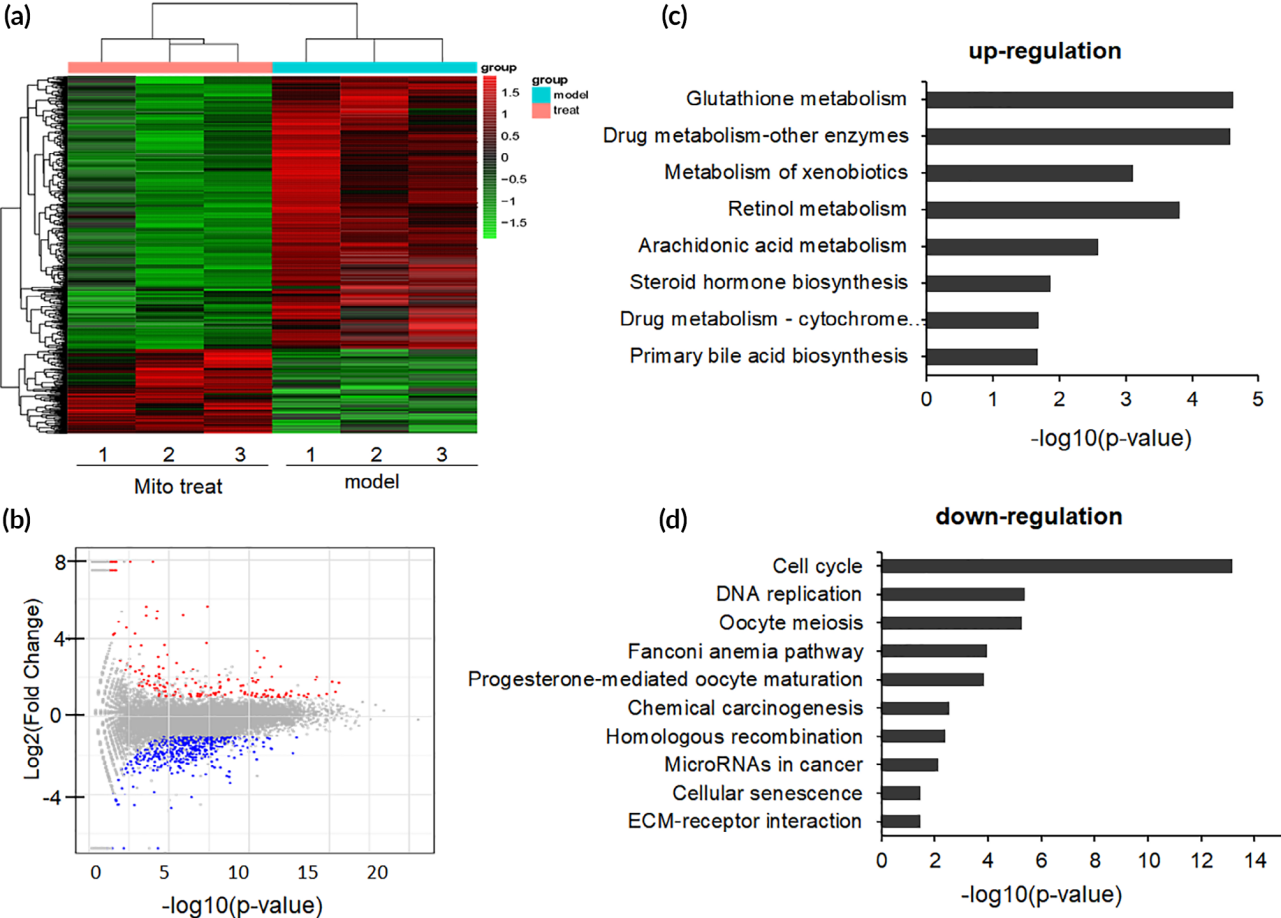
Enriched KEGG pathways after mitochondrial therapy. (a) Heatmap for proteins and enzymes were showed in mouse liver tissues of CCl_4_‐induced injury and mitochondrial treatment group (mitochondrial dosage 0.4 mg/kg body weight), values were represented by log10 (fold changes). (b) A summary of the numbers of up‐ and down‐regulated DEGs for each GO subcategory. (c) The up‐regulated eight significantly enriched KEGG pathways and (d) the down‐regulated 10 KEGG pathways. The pathways were generalized according to *p* < 0.05

Further, KEGG cellular pathways were executed to sort out the canonical signaling pathways of DEGs. A sum of 1055 DEGs (195 up‐regulated genes and 860 down‐regulated genes) were corresponded to the simple pathway mapping and established as 259 KEGG pathways, and the DEGs were categorized into 20 statistically consequential classes (*p* < 0.05, Figure 6a,b) and were predominantly refined into six main pathways: metabolism (12), organismal systems (5), human diseases (4), cellular processes (4), genetic information processing (4), and environmental information processing (1). Most of the up‐regulated DEGs were associated with xenobiotic metabolic activity and hepatoprotective mechanism (Figure 6c). However, the majority of down‐regulated DEGs were tightly correlated with cell cycle arrest (Figure 6d).

### 2.6 Mitochondria activated the anti‐oxidant system

CCl_4_ is known to produce reactive free radicals and initiation of cell damage through conjugation of the free radicals to the membrane proteins. CCl_4_ and the generated ROS are associated with impaired glutathione metabolism, finally counteracting the anti‐oxidant effects. Also, increasing ROS simulate a vital role in the production of the liver injury and initiation of the hepatic fibrogenesis. However, the cellular anti‐oxidant system was highly activated by the mitochondrial administration. From the KEGG pathway, glutathione metabolism was significantly up‐regulated by mitochondria. In addition, a series of genes of anti‐oxidant enzymes, including *PDH*, *GPx*, *PON*, *SOD*, *CAT*, and *TXNR*, was transcriptionally up‐regulated (Table S1). The activation of these enzymes states that an enormous quantity of ROS was eliminated from the liver after the mitochondrial treatment.

**FIGURE 7:**
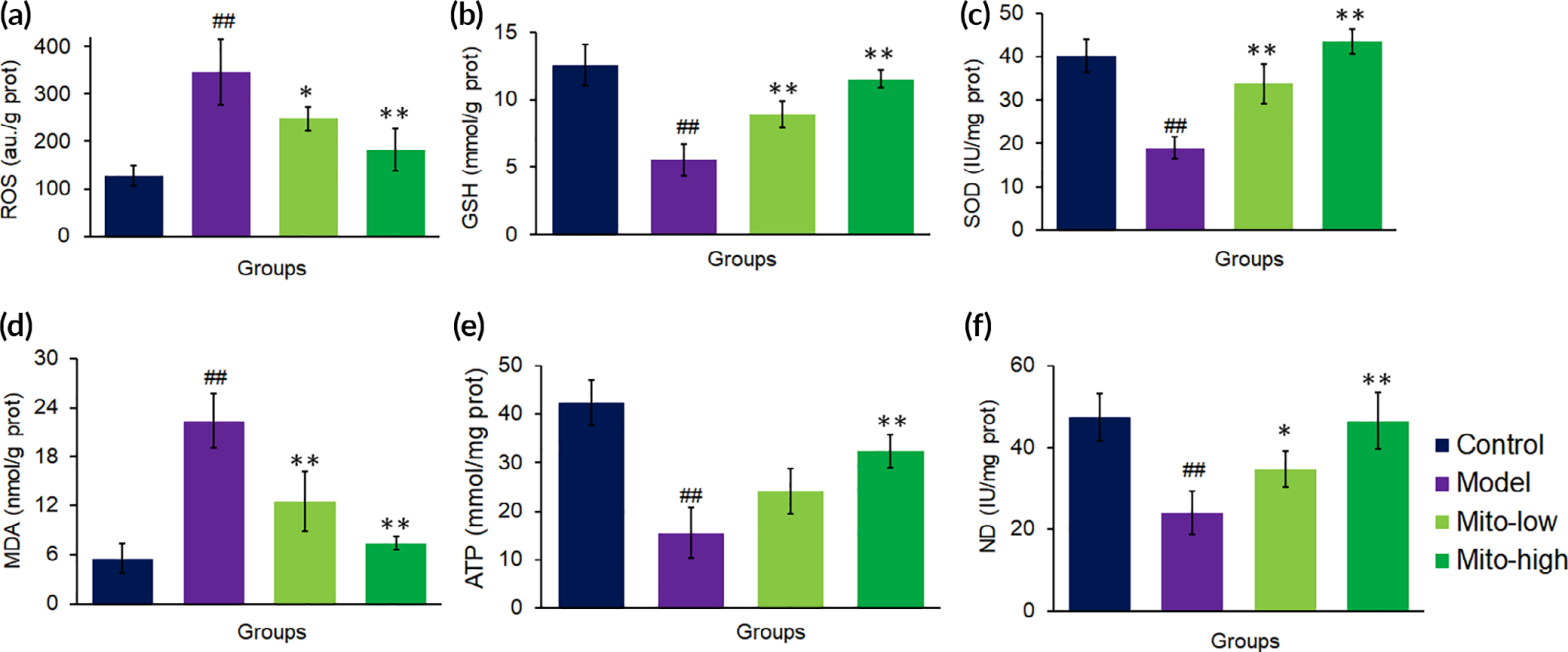
Mitochondria diminished ROS level and increased ATP supply in mouse liver tissues. The redox system were measured, including (a) ROS, (b) GSH, (c), SOD and (d) MDA. Moreover, the levels of (e) ATP content and (f) ND activity were determined, respectively. Each group contained eight mice (*n* = 8 for each group). ^##^ *p* < 0.01 compared with the control; *p < 0.05, **p < 0.01 compared with the CCl_4_ group

The result was further identified by biochemical assay, in which ROS level significantly reduced from 412.98 to 241.40 a.u/gprot (mito‐low group) and 152.24 a.u/gprot (mito‐high group), respectively, while GSH content increased from 5.51 to 8.91 mmol/gprot (mito‐low group) or 11.50 mmol/gprot (mito‐high group) after the mice were donated with the exogenous mitochondria (Figure 7a,b). Meanwhile, SOD activities increased, and MDA reduced after mitochondria treatment (Figure 7c,d), indicating that the mitochondria could diminish the oxidative stress caused by CCl_4_.

### 2.7 Mitochondria restored oxidative phosphorylation function

CCl_4_ can impair the respiratory chain of liver mitochondria and induce the marked respiratory inhibition that leads to a decrease of ATP generation. However, a series of oxidative phosphorylation (OXPHOS)‐related genes, including *Coq10a*, *Ndufaf1*, *Fmc1*, *Ndufs7*, *Sdhb*, *Mrpl9*, and *Atp5d*, are up‐regulated to rebuild the respiratory chain and ATP synthase (Table S2). Notably, the genes of mitochondria‐coded ATP synthase 6 (*Mt‐ATP6*) and COX2 subunit (*mt‐Nd2*) were also activated. The up‐regulated OXPHOS‐associated genes would result in the increases of ND activity and ATP production after mitochondria treatment, which was identified by the biochemical measurements (Figure 7e,f).

**FIGURE 8:**
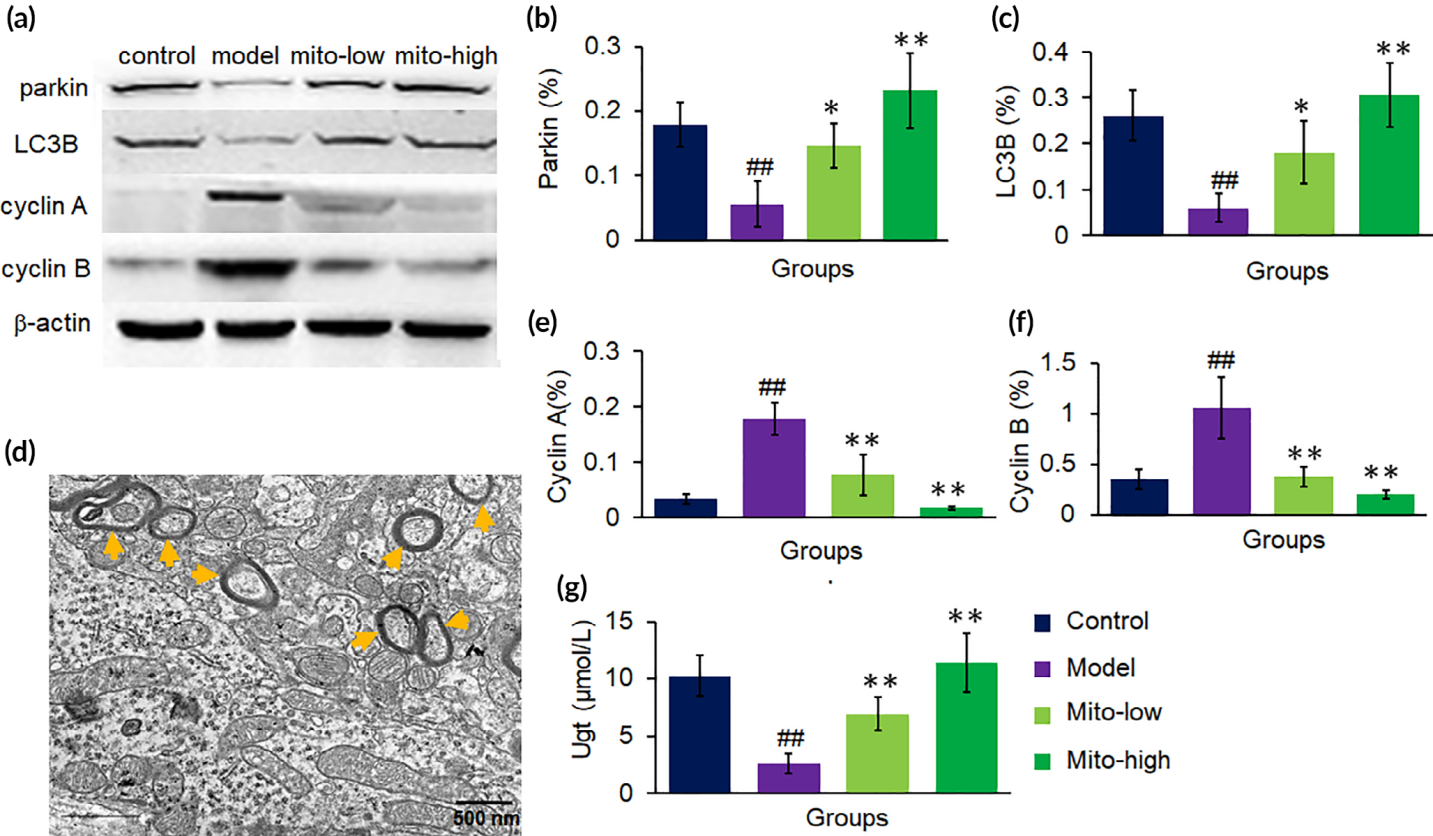
Representative proteins in transcriptomic analysis were further examined by WB in mouse liver tissues. (a) WB bonds of parkin, LC3B, cyclin A and B. (b and c) Respective ratio of the gray value of autophagy proteins, parkin and LC3B, to β‐actin. (d) A representative picture showed numbers of autophagosome appeared after mitochondrial treatment. The sections were observed under TEM. (e and f) Ratios of the gray value of cyclin A or B, to β‐actin. (g) Ugt level. *N* = 6 for each group. ^##^ *p* < 0.01 compared with the control; *p < 0.05, **p < 0.01 compared with the CCl_4_ group

Moreover, the mitochondria activated autophagy‐related gene transcription (*Gabarapl1*, *LC3b*, *Atg*, *Prkn*, *Pink1*) (Table S3) and expression (Figure 8a,c). It is reported that autophagy in hepatocytes is reduced in CCl_4_‐induced liver damage,^16^, ^17^ which leads to the broken organelles and damaged proteins accumulated in cells to secondarily destroy hepatocytes. However, mitochondrial therapy promoted autophagy, and then the organelles would be degraded to provide ATP for cellular homeostasis. In addition, PINK1 and parkin play an essential role in mitophagy, which are closely associated with mitochondrial health and quality control. Under TEM, mitophagy obviously increased after mitochondrial administration (Figure 8d). The dysfunctional mitochondria caused by CCl_4_ would be eliminated by mitophagy, and then the healthy mitochondria would be responsible for energy supply and maintain cell function.^18^, ^19^

### 2.8 Mitochondria prevented cell proliferation

The biology of acute liver injury is characterized by hepatocellular damage and inflammation. Thus, cell cycle arrest represent a vital role in the prevention of hepatocytes and maladaptive repair following hepatocellular injury, avoiding replication of damaged DNA and carcinogenesis.^20^ In this study, an extensive range of genes associated in the cell proliferation were recognized to be considerably inhibited by the mitochondria and most of them belonged to the ZH‐C2H2 transcription factor‐mediated cascade system (Table S4), and the representative cell cycle regulators, cyclin A and B, were significantly down‐regulated, evaluated by WB analysis (Figure 8a,e,f). The cell cycle arrest induced by the cascade system will prevent the gathering of DNA mutations that propagate into cancer.

### 2.9 Mitochondria accelerated the xenobiotic metabolism transformation

Since CCl_4_ and its metabolites can induce hepatocyte injury, drugs that accelerated the xenobiotic metabolism would protect the cells. In the liver, cytochromes P450 (Cyp), UDP‐glucuronosyltransferases (UGT), glutathione S‐transferase (GST), and N‐acetyltransferase (NAT) are accountable for metabolic process of xenobiotics and are required for the efficient elimination of chemicals from the body. UGT is an important transferase which forms conjugates of glucuronyl group with an extensive range of lipophilic substrates producing hydrophilic glucuronide conjugates that are excreted from the body. Thus, glucuronidation exhibits an essential detoxification mechanism for xenobiotics, such as drugs and harmful industrial chemicals. In this study, genes of *Cyp*, *UGT*, *GST*, and *Nat* were significantly up‐regulated after mitochondrial treatment (Table S5), in which UGT level was dramatically increased in the mitochondria‐treated group compared to the model mouse liver (Figure 7g), suggesting that the mitochondria facilitated the transformation and elimination of CCl_4_.

### 2.10 Mitochondria maintained liver protein homeostasis

After hepatocyte damage occurred, globulin production in the liver will decrease, and protein homeostasis (proteostasis) will be damaged. Among specific proteins synthesized by the liver, major urine proteins (MUPs) are types of α_2u_‐globulin analogous protein and are produced in large amounts in healthy male mouse liver that contains 30,000 copies of MUP mRNA per cell. MUPs' family members possess a conserved β‐barrel framework with a featured central hydrophobic region. MUPs can encapsulate lipophilic molecules into their pockets and are secreted in the kidney. Moreover, MUPs are unique members of the lipocalin superfamily that mediate metabolic signaling to maintain normal hepatocyte function. Transcriptome analysis showed that a series of genes encoding MUPs, including *Mup11*, *Mup20*, *Mup19*, *Mup18*, *Mup16*, *Mup12*, is activated after mitochondrial treatment (Table S6). The escalated MUPs might bind to CCl_4_ in the central hydrophobic pocket and reduce its toxicity to the liver.

### 2.11 Molecular signal transduction extrapolation

**FIGURE 9:**
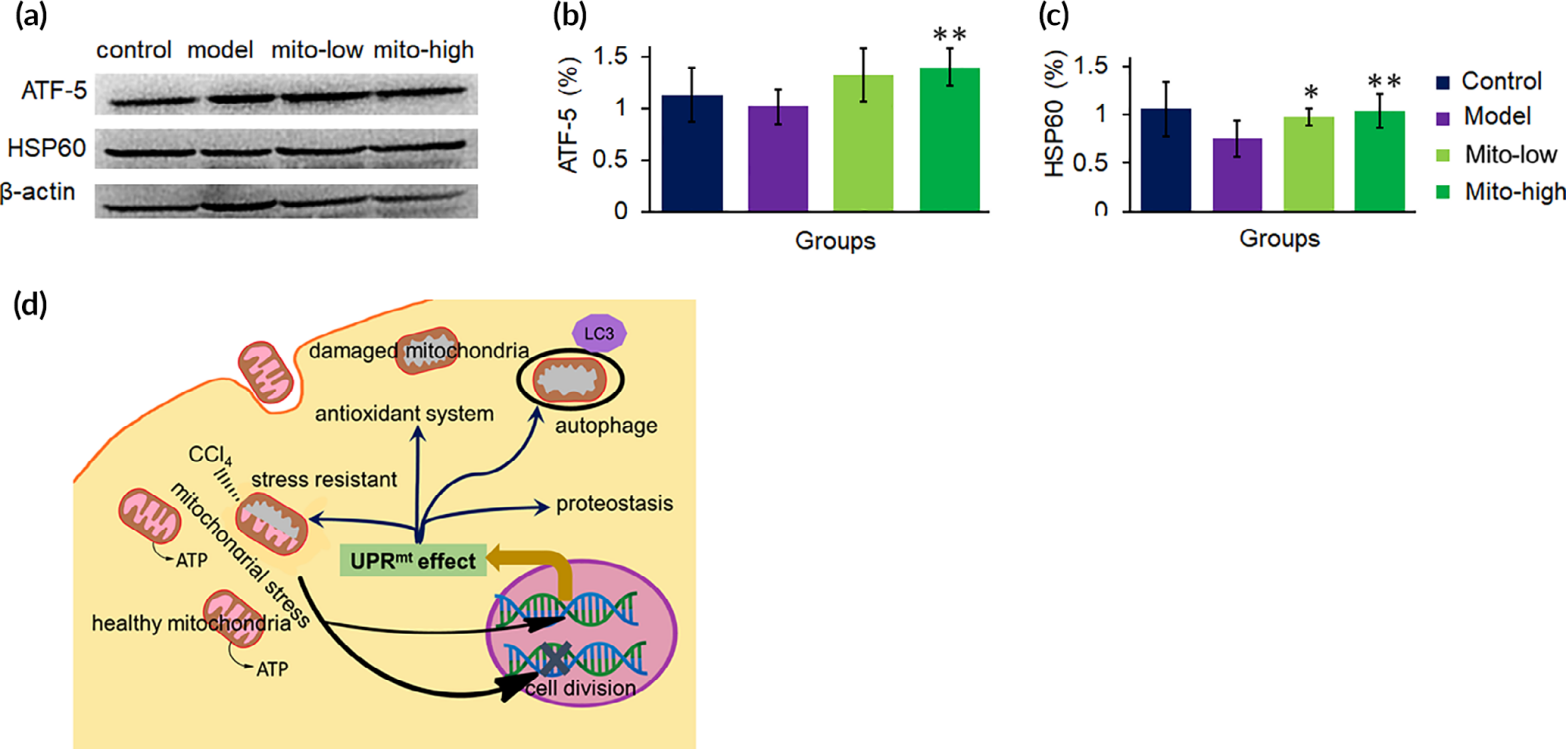
Molecular signal pathways involved in mitochondrial therapy on CCl_4_ induced liver injury. (a) WB bonds of ATF5 and HSP60. (b and c) Respective ratio of the gray value of ATF‐5 and HSP60 to β‐actin. (d) The mechanism of mitochondrial therapy. Mitochondria stress was induced once entering cells, and then activated the UPR^mt^ pathway, a mitochondria‐nuclear retrograde signal, to promote gene transcription and expression of a series of protective proteins and enzymes, including anti‐oxidant enzyme, OXPHOS‐related proteins, biotransformation enzymes, proteostasis, and autophagy‐related proteins

In order to frame out the all relevant, effective cell signaling pathways produced by the mitochondria, we outlined the gene regulatory network frame through mapping of the RNA‐seq data and the existing DNA‐protein interaction database and protein–protein interplay (Cytoscape 3.5.0 software). Mitochondrial unfold protein reaction (UPR^mt^)‐induced gene transcription was recognized to be the most pertinent factor in a sequence of biological activities from anti‐oxidants, OXPHOS, xenobiotic biotransformation, proteostasis, and autophagy. The extrapolation was supported by the up‐regulation of gene transcription of UPR^mt^ molecular markers, including *Dnaj*, *HSPs*, *lonp1*, *Clpp*, *atf5* (Table S7). Also, the levels of two representative proteins of UPR^mt^, activating transcription factor 5 (ATF‐5) and heat shock protein 60 (HSP60), were respectively determined by WB, and the result identified that the protein levels elevated after mitochondrial administration (Figure 9a–c). Collectively, we assume that mitochondria may be attacked by CCl_4_ after entering liver cells, which would cause mitochondrial stress, and then induce cell cycle arrest and meanwhile promote the UPR^mt^ protective mechanism (Figure 9d). Further resulting in improving the activity of anti‐oxidant enzymes to diminish ROS, increasing autophagy to eliminate damaged mitochondria and organelles, and enhancing OXPHOS to increase ATP production. The signal pathways could explain the molecular mechanism of mitochondria on cell protection.

## 3 DISCUSSION

CCl_4_ is a toxic industrial chemical that causes acute and chronic active hepatitis. Hepatic mitochondrial functions are impaired in animals due to CCl_4_ hepatotoxicity. Mitochondrial transplantation therapy enhances the energy supply to hepatocyte cells and reduces the oxidative stress, leading to increased recovery of cell viability. The molecular mechanisms underlying the efficacy of the mitochondrial therapy were associated with the activation of the URP^mt^ signal pathway to recover cell homeostasis, which are important in preserving hepatocyte function.

Mitochondria are the organelles of micro‐nano size (0.5–1 μm). It is known that macropinocytosis and cathrin‐mediated endocytosis may be the entry pathway of nanocapsules in micro‐nano size. To clarify the cell entry mechanism of mitochondria, we used the inhibitors to investigate the internalization pathway of the mitochondria. The result showed that macropinocytosis could be the plausible entry pathway because macropinocytosis inhibitor amiloride inhibits the mitochondrial internalization. The result is consistent with the report that DsRed‐ and GFP‐tagged mitochondria cannot enter cells with amiloride‐treated cells.^21^

After the mitochondria enter cells, they are transported to lysosomes. Majority of them escape from the lysosomes and play roles in cytosol. ^22^ Then the mitochondria exhibit the ability to increase ATP production, activate the anti‐oxidative system and reduce the ROS level, which leads to cell viability increase and functional recovery in a certain concentration range.^23^, ^24^ Recent reports also suggest that mitochondrial transplantation therapy can lead to enhanced bioenergetics in normal cardiomyocytes, and meanwhile there is no increase in superoxide production.^25^ However, only ATP administration does not show improvement in cardiac function, which implies that mitochondrial therapy could induce tissue regeneration.^26^ This advantageous ability of the mitochondria has been utilized in the treatment of mitochondria‐related diseases, including myocardial ischemia, cerebral ischemia, schizophrenia, depression, aging, and tumor.^21^, ^27^ In the study, the elevated serum transaminase activities, and the decreased GSH and SOD levels that is intoxicated with CCl_4,_ were predominately stopped by the exogenous mitochondrial administration. Also, the mitochondria prevented liver fibrosis and retained the ultracellular structure of the hepatocytes.

Since molecular signal mechanism of mitochondrial therapy is still unclear, we used transcriptomic analysis to address the issue in the study. The results suggest that the mechanism of the mitochondrial therapy on CCl_4_‐induced hepatocyte damage is closely associated with the UPR^mt^‐regulated pathway, a protective transcriptional response mediated by mitochondria‐to‐nuclear retrograde signaling.^28^, ^29^ UPR^mt^ can be activated by mitochondrial stress that is induced by mitochondria unfold proteins, pathogens, and toxins,^30^ then significantly increased expression of a broad of UPR^mt^‐regulated genes, including HSPs, respiratory chain proteins, homeostasis proteins (proteostasis), detoxification response (ROS defense and toxin detoxification), autophagy, protease, and metabolism‐associated proteins.^31^, ^32^, ^33^ Activation of the UPR^mt^ promotes the anti‐oxidant gene expressions to stabilize mitochondrial function and increase adaptation in mitochondrial stress, which are linked to healthy lifespan and longevity. At present, UPR^mt^ is recognized as a protective mechanism that allows the synchronization of nuclear and mitochondria to maintain cell homeostasis.^34^, ^35^

Among the UPR^mt^‐regulated genes, anti‐oxidant genes containing *PON*, *SOD*, and genes involved in GSH metabolism is activated to prevent the protein and membrane injury induced by free radicals.^36^ In this study, the elevated anti‐oxidant system will stabilize the intracellular environment and recover functions of salvageable organelles damaged by CCl_4_, and the irreparable organelles could be eliminated via autophagy activated by UPR^mt^. In addition, UPR^mt^ can up‐regulate the expression of xenobiotic detoxification genes such as UGTs and GST, which would accelerate CCl_4_ excretion from the body to prevent its hepatotoxicity.

CCl_4_ is a commonly used toxin to cause liver injury since it can induce free radicals and trigger peroxide chain reaction, leading to lipid peroxidation in phospholipid‐rich and membrane structures. Mitochondria contain phospholipid bilayers, and especially the inner membrane is the main site of cellular energy production (OXPHOS).^5^ The free radicals damage the capability of OXPHOS and decrease ATP production. Moreover, free radicals can impair proteins and nucleic acids, and then aggravate cell injury. Thus, CCl_4_‐induced liver injury could be prevented through scavenging free radicals and restoring cellular energy supply.^37^, ^38^ Here we used the mitochondrial transplantation therapy to deliver healthy mitochondria into mice. The exogenous mitochondria have intact membrane structures, which can recover energy supply. Also, the mitochondria can activate cell protective UPR^mt^ effect, then promote anti‐oxidant system to scavenge free radicals. In addition, the activated autophagy could eliminate the damaged mitochondria and other organelles. Therefore, administration of healthy mitochondria improve hepatocyte function that impaired by CCl_4_.

Nevertheless, the mechanism of the mitochondrial therapy on cell cycle arrest is still unknown. Increasing evidence shows that mitochondrial function is closely associated with cell cycle.^39^, ^40^ One of evidence comes from the study of yeast mitochondrial inheritance.^41^ The OXPHOS capability of *Saccharomyces cerevisiae* decreases when the cells enter into mitosis, while the capability is recovered during meiosis, and then decreased again at the post‐tetrad stage before budding, implying that increase of OXPHOS might induce cell cycle arrest. A recent report shows that activation of transcription factor daf‐16 (FOXO orthologue in mammals) after the UPR^mt^ is activated in *Caenorhabditis elegans*, in which FOXO is an important cell regulator to induce cell cycle arrest and DNA repair.^32^, ^42^ During liver injury caused by CCl_4_, cell cycle arrest in hepatocytes and nonparenchymal cells would benefit the cell repair from injury at the resting phase, and prevent the progression of liver fibrosis.^43^, ^44^

Here we used intact mitochondria to study their function on CCl_4_‐induced liver injury through the intravenous administration. After the mitochondria are injected into blood vessels, they would increase the content of mitochondria in blood. It is known that blood contains intact cell‐free mitochondria in both healthy people and patients, which would be important in maintaining normal physiological functions. ^45^, ^46^ At present, a new concept has arisen recognizing that extracellular mitochondria are important players in tissue regeneration and immune regulation,^47^ which will benefit the recovery of liver function that damaged by CCl_4_. Nevertheless, the effects of intact mitochondria on liver cells (including hepatic stellate cells and kuffer cells) and blood immune cells will continue to be further investigated.

## 4 CONCLUSIONS

Today mitochondria are regarded as more than an energy plant in cells. Mitochondria are signaling organelle that maintain cell homeostasis and elicit tissue repair and regeneration. The mitochondrial therapy with protective purposes have made rapid progress from cell and animal studies to clinical trials. ^48^ Isolated mitochondria can enter cells after simply co‐incubation, then play beneficial roles through increasing bioenergy supply and diminishing free radicals. Mitochondrial therapy also prevents liver cell injury in animals with CCl_4_ intoxication by activating the signal transduction to nuclear and induce various gene transcription and expression. The protective response reconstructs the stable balance of mitochondria, and intimately enhance stress resistance that is linked to cell survival. The study provides a novel approach for preventing liver injury caused by toxins and suggests that activation of mitochondria‐unclear signal pathway would be the molecular mechanism the mitochondrial therapy.

## 5 MATERIALS AND METHODS

### 5.1 Animals

Healthy Kunming mice (SCXK [Jing 2006–2009]), weight ranging from 18 to 22 g, were utilized for the research. The mice were procured from Chongqing Medical University, China. The mice were sheltered in the SPF center and fed using high‐quality mouse chow and clean water. The animal procedure was authorized by the Animal Ethical Committee, Southwest University.

### 5.2 Mitochondrial isolation and staining

Liver mitochondria from healthy mice were isolated and activity determination according to the earlier reports.^14^, ^24^, ^49^ In brief, the mice were euthanatized through quick cervical dislocation. Further, the mouse liver was removed and put in ice‐cold PBS (pH 7.4). The liver was frozen and homogenized (0–4°C) and further centrifuged at a rate of 800*g* for 5 min. Then the supernatant fraction was obtained and added into the isolated mitochondrial solution. The suspension was further centrifuged at 10,000*g* for about 10 min, and the precipitate was re‐suspended in the isolated solution and centrifuged again. The obtained mitochondria were stained using the mitochondrial specific indicator, Mitotracker red CMXRos (0.1 μmol/L, Invitrogen, Cambridge, MA) was utilized based on the manufacturer's protocol. Mitotracker Red CMXRos produces covalently bonded conjugates with the functional thiol group of proteins in the mitochondria. The mitochondria were placed on slides and observed through a fluorescence microscope (Olympus Corporation, Tokyo, Japan) at 575 nm excitation wavelength and 600 nm emission wavelength. In addition, mitochondrial number was counted under an optical microscope (Olympus, Tokyo, Japan), and the concentration was examined by the BCA assay.

### 5.3 Cell culture

Mouse hepatocytes were cultured according to our earlier report (Shi et al.^14^). The cells were preserved in Dulbecco's modified Eagle's medium (DMEM) complemented with 10% FBS at constant temperature and sterile culture incubator along with 5% CO_2_ at 37°C (ESCO, Indonesia). All media were obtained from Gibco.

### 5.4 Cell internalization of mitochondria

To elucidate the mechanism of cell entry of the isolated mitochondria, macropinocytosis inhibitor amiloride and cathrin‐mediated endocytosis inhibitor chlorpromazine, were respectively put on into the cell media. After 30 min, mitochondria were introduced into the DMEM and further incubated for another 4 h. The cells were further cleaned using PBS for 3 times. Nucleus was stained by DAPI. Fluorescence was observed, and images were obtained with a confocal microscope (Zeiss LSM 510, Germany).

### 5.5 CCl_4_‐induced cell injury and biochemical assay

When the cells grew up, CCl_4_ (10 mM) were introduced inside cultured cell media and incubated for about 12 h. Further, the media was substituted by fresh DMEM, and various different concentrations of exogenous healthy mitochondria were introduced inside the cell culture medium. According to mitochondrial number and protein level determination, mitochondria of 1 mg/ml was equivalent to 1.6 × 10^8^ mitochondria per ml. Cell viability was estimated by utilizing AlamarBlue® Cell Viability Assay Reagent (Pierce, USA), based on the protocol of the manufacturer. The absorbance of the cells was obtained at 630 nm on the microplate reader. Cell media without mitochondria treatment were utilized as a control sample. The relative cell viability was determined as cell viability (%) = OD (blank‐sample)/OD (blank‐control). Moreover, ATP level and quantity, amount of reactive oxygen species (ROS) produced, glutathione (GSH) level, superoxide dismutase (SOD), malondialdehyde (MDA), and NADH dehydrogenase (ND), were individually calculated using commercial kits (Nanjing Jiancheng Biotech. Ltd., Co., Nanjing, China). Six isolated experiments were conducted for every individual assay.

### 5.6 In vivo biodistribution analysis

Fluorescence imaging of the cells in vivo condition was obtained utilizing an In‐Vivo Imaging System (BLT Photon Tech., Guangzhou, China). The mice were administered with PBS solution with 10^8^ Mitotracker‐labeled mitochondria through the tail veins. After 2 h of administration of mitochondria, the mice were strongly anesthetized using 3% sodium pentobarbital, and further transcardial insertion of PBS (0.01 M, pH 7.4) was done to separate the blood of the mouse. The organs of the mouse, such as the lung, kidney, liver, heart, were removed and set out in the cassette. Images of the organs were obtained using fluorescence devices for the mice served with the dosage of Mitotracker‐labeling in the mitochondria. The images were overspread based on the procedure followed by the manufacturer. Further, the tissues of the mice were settled using 4% paraformaldehyde dissolved in the PBS solution. The frozen parts (30 μm) of tissues were individually pierced with a cryomicrotome (Leica, Germany), and the frozen parts fluorescence was examined under the confocal microscope.

### 5.7 Mitochondrial treatment of CCl_4_‐induced mouse liver injury

Animal model of liver injury was prepared by using 3‐week treatment of adult male mice with 20% CCl_4_ [CCl_4_ in olive oil (1:4, v/v), 1 μl per kg body weight by subcutaneous injection once in 3 days]. After 3 weeks of CCl_4_ treatment, the CCl_4_‐injected mice were randomly designated into three groups (*n* = 10 in each group). Mice in mitochondrial therapy group were respectively intravenously (i.v.) injected with 0.2 mg/kg (Mito‐low) or 0.4 mg/kg (Mito‐high) mitochondrial solution in saline once every day for 7 days. The model mice have injected the equal volume saline. To prepare the mouse model of liver fibrosis, the CCl_4_ was administrated continuously for 5 weeks. Then the mice were given the mitochondrial treatment. The controls mice (*n* = 10) received olive oil only.

### 5.8 Biochemical assay and tissue evaluations

Serum levels of alanine aminotransferase (ALT) and aspartate aminotransferase (AST) were respectively estimated by standard procedures of automatic biochemistry analyzer. Hydroxyproline content was estimated using the Jamall methods as earlier reported. UDP‐glucuronosyltransferase (UGT) level was measured according to the commercial kit (Shanghai Jianglai industrial Co., Ltd., China). Moreover, liver tissues were settled in the 10% buffered formalin and sectioned on a paraffin microtome. Then the sections of the liver tissues were stained using hematoxylin–eosin (HE) or Sirius red staining.

### 5.9 Liver mitochondrial activity assay

To measure mitochondrial activity in vivo after mitochondrial treatment. Mitochondria from mouse liver were extracted 24 h after the exogenous mitochondrial injection. The activities of the extracted mitochondria were respectively measured. Also, TEM was used to observe the mitochondrial morphology.

### 5.10 Western blot analysis

Protein extraction of liver specimens was exposed to SDS‐PAGE and transmitted onto PVDF membrane, further investigated with antibodies versus LC3B, parkin, cyclin A and B, ATF‐5, HSP60 and β‐actin (Beijing boason Biotech. Co., Ltd., Beijing, China), then with HRP‐conjugated secondary antibodies (1:5000; Beijing Dingguo Biotech. Co., Ltd., Beijing, China) as the secondary antibody. After the membranes were washed twice for about 15 min each using wash buffer, the signal was identified by the ECL system (Pierce Co.).

### 5.11 RNA extraction, library preparation, and Illumina Hiseq4000 sequencing

The whole RNA of mouse liver (100 μg/ml) was acquired utilizing TRI Reagent® based on the manufacturer's procedure (Sigma‐Aldrich, Co.). The quality of the RNA was estimated utilizing the Agilent 2100 Bioanalyser system (Agilent, USA) and quantified utilizing the NanoDrop ND‐2000C (Thermo Scientific, USA). The RNA samples of High‐quality standard (OD260/280 = 1.8–2.2, OD260/230 ≥ 2.0) were utilized to build the cDNA library. Transcriptomic RNA‐seq libraries were produced following the guidelines of TruSeqTM RNA sample production Kit from Illumina (San Diego, CA, USA). Further, mRNA was extracted using polyA collection from oligo(dT) beads and fragmented into pieces using fragmentation buffer. The cDNA synthesis, A‐base addition, end repair, and ligation of the Illumina‐indexed adaptors were conducted based on the Illumina's procedure. Libraries of the RNA were further size segregated for obtaining targeted cDNA fragments and then continued by PCR amplification utilizing Phusion DNA polymerase (NEB, England, UK) for about 15 PCR cycles. Further assessed by TBS380 and the end‐paired RNA libraries were further sequenced by the Illumina HiSeq (2 × 150 bp read length).

### 5.12 Read mapping and sequence assembly

The untreated raw and pure paired‐end reads of the sequence were cut, trimmed, and the sequence quality was controlled by SeqPrep and Sickle with revert existing parameters. Further, the alignment of pure reads was uniquely maintained based on the reported genome reference utilizing orientation mode with Bowtie2 software. Further particular zone of the gene was expanded according to subsequent depths of sites, and then the operon was acquired. In supplement to this, further, the whole genome of RNA was further split into numerous fragments of 15 kb windows, which serve 5 kb each. The newly transcribed zone was stipulated as additional two successive windows without the overlapped zone of the gene, where a minimum two reads where mapped per window in the identical orientation.

### 5.13 Differential expression analysis and functional enrichment

Further, for recognizing the differential expression genes (DEGs) among the two types of the sample groups, the level of expression of the genes for each and every transcript was determined to utilize the fragments per kilobase of exon per million mapped reads (FRKM) methodology. Cuffdiff was utilized to study the primary differential gene expression, and the examination of DE‐seq is conducted through R software was then initiated to test numerous DEGs. The DEGs among exogenous mitochondrial administration therapy in the sample groups and control groups were recognized utilizing the upcoming criteria: (1) the logarithmic value of fold change should be higher than 2.5, (2) the FDR range should be lower than 0.05. Further, to estimate the function of the DEGs, gene ontology (GO) functional enhancement and KEGG pathway examination were performed by Goatools and KOBAS tools. DEGs were predominantly enhanced in GO terms and cellular metabolic pathways when Bonferroni‐corrected *p* < 0.05.

### 5.14 Statistical analysis

The collected data were demonstrated as mean ± SD for every sample. Numerous comparisons among the control groups and other groups were examined by Dunnett's test. Differences were observed considerable when *p* < 0.05.

## AUTHOR CONTRIBUTIONS

**Zizhen Zhao:** Investigation; methodology; writing‐original draft. **Yixue Hou:** Formal analysis; methodology; validation. **Wei Zhou:** Formal analysis; methodology; validation. **Keerthiga Rajendiran:** Writing‐original draft. **Ailing Fu:** Data curation; funding acquisition; supervision; writing‐review and editing.

## CONFLICT OF INTEREST

The authors declare that there is no conflict of interest that could be perceived as prejudicing the impartiality of the research reported.

### PEER REVIEW

The peer review history for this article is available at https://publons.com/publon/10.1002/btm2.10209.

## Supporting information


**Table S1**: Mitochondria activated anti‐oxidant system of cells.
**Table S2**: Mitochondria up‐regulated OXPHOS‐related enzymes and proteins in the respiratory chain.
**Table S3**: Mitochondria increased autophagy‐associated gene transcription.
**Table S4**: Mitochondria down‐regulated cell cycle‐associated gene transcription.
**Table S5**: Mitochondria increased gene transcription of metabolic enzymes and protein
**Table S6**: Mitochondria up‐regulated gene transcriptions of a series of MUPs.
**Table S7**: Mitochondria up‐regulated gene transcriptions of UPR^mt^ markers.

## Data Availability

The data that support the findings of this study are available from the corresponding author upon reasonable request.
