# Supplementary material for: Mitochondrial transplantation therapy inhibit carbon tetrachloride‐induced liver injury through scavenging free radicals and protecting hepatocytes
Source: Bioeng Transl Med. 2020 Dec 30;6(2):e10209. doi: 10.1002/btm2.10209 (PMC8126821; doi:10.1002/btm2.10209)
Supplement: Supplementary file 1 — Table S1: Mitochondria activated anti‐oxidant system of cells. Table S2: Mitochondria up‐regulated OXPHOS‐related enzymes and proteins in the respiratory chain. Table S3: Mitochondria increased autophagy‐associated gene transcription. Table S4: Mitochondria down‐regulated cell cycle‐associated gene transcription. Table S5: Mitochondria increased gene transcription of metabolic enzymes and protein Table S6: Mitochondria up‐regulated gene transcriptions of a series of MUPs. Table S7: Mitochondria up‐regulated gene transcriptions of UPRmt markers. [file BTM2-6-e10209-s001.docx]

**Supplementary data**

**Table S1.** Mitochondria activated anti-oxidant system of cells.

| **gene name** | **log2foldchange** | **p-value** | **E.C.** | **description** |
| --- | --- | --- | --- | --- |
| *Prodh* | 1.76 | 8.57E-21 | EC:1.5.5.2 | proline dehydrogenase |
| *GPx* | 1.17 | 4.36E-11 | EC:1.11.1.9 | glutathione peroxidase |
| *PON1* | 1.12 | 2.87E-10 | EC 3.1.8.1 | paraoxonase1 |
| *Csrp3* | 2.43 | 1.55E-10 | - | cysteine and glycine-rich protein 3 |
| *SOD2* | 0.53 | 3.25E-03 | EC:1.15.1.1 | superoxide dismutase 2, mitochondrial |
| *SOD1* | 0.48 | 7.16E-03 | EC:1.15.1.1 | superoxide dismutase 1 |
| *CAT* | 0.55 | 9.44E-03 | EC:1.11.1.6 | catalase |
| *TXNR* | 0.44 | 3.77E-02 | EC:1.8.1.9 | thioredoxin reductase 1 |

**Table S2.** Mitochondria up-regulated OXPHOS-related enzymes and proteins in the respiratory chain.

| **gene name** | **log2foldchange** | **p-value** | **E.C.** | | **description** | |  |
| --- | --- | --- | --- | --- | --- | --- | --- |
| *Coq10a* | 0.91 | 4.47E-05 | | - | | coenzyme Q10A | |
| *Mt-ATP6* | 1.65 | 1.55E-04 | | EC 3.6.1.3 | | mitochondrially encoded ATP synthase 6 | |
| *Clpx* | 0.62 | 5.67E-04 | | - | | caseinolytic mitochondrial matrix peptidase chaperone subunit | |
| *Mtfr2* | -2.14 | 7.75E-04 | | - | | mitochondrial fission regulator 2 | |
| *Ndufaf1* | 0.51 | 1.30E-03 | | - | | NADH:ubiquinone oxidoreductase complex assembly factor 1 | |
| *mt-Nd2* | 0.40 | 8.97E-03 | | EC 1.6.5.3 | | mitochondrially encoded NADH dehydrogenase 2 | |
| *Fmc1* | 0.79 | 2.65E-03 | | - | | mitochondrial complex V assembly factor | |
| *Ndu* | 0.53 | 5.35E-03 | | EC1.6.5.3 | | NADH:ubiquinone oxidoreductase | |
| *Sdh* | 0.48 | 8.04E-03 | | EC:1.3.5.1 | | succinate dehydrogenase complex | |
| *Mrpl9* | 0.53 | 8.31E-03 | | - | | mitochondrial ribosomal protein L9 | |
| *Atp5d* | 0.38 | 3.37E-02 | | EC 3.6.1.34 | | ATP synthase, H+ transporting, mitochondrial F1 complex | |

**Table S3.** Mitochondria increased autophagy-associated gene transcription.

| **gene name** | **log2foldchange** | **p-value** | **EC** | **description** |
| --- | --- | --- | --- | --- |
| *Gabarapl1* | 0.95 | 9.67E-08 | - | gamma-aminobutyric acid (GABA) A receptor-associated protein-like 1 |
| *Map1lc3b* | 0.82 | 4.63E-05 | - | microtubule-associated protein 1 light chain 3beta |
| *Atg101* | 0.68 | 5.12E-03 | - | autophagy related 101 |
| *Prkn* | 0.40 | 2.86E-02 | EC:2.3.2.31 | parkin RBR E3 ubiquitin protein ligase |
| *Atg5* | 0.40 | 3.20E-02 | - | autophagy related 5 |
| *Atg2a* | 0.41 | 3.41E-02 | - | autophagy related 2A |
| *Pink1* | 0.53 | 3.69E-02 | EC:2.7.11.1 | PTEN induced putative kinase 1 |

**Table S4.** Mitochondria down-regulated cell cycle-associated gene transcription.

| **gene name** | **log2foldchange** | **p-value** | | **EC** | | **description** | |  |
| --- | --- | --- | --- | --- | --- | --- | --- | --- |
| *Ccnb1* | -2.48 | | 2.88E-19 | | - | | cyclin B1 | |
| *Ccnb2* | -2.04 | | 2.45E-16 | | - | | cyclin B2 | |
| *Cdc20* | -1.88 | | 9.13E-16 | | - | | cell division cycle 20 | |
| *Ccna2* | -2.13 | | 1.26E-13 | | - | | cyclin A2 | |
| *Bub1* | -1.90 | | 4.51E-10 | | EC:2.7.11.1 | | mitotic checkpoint serine/ threonine kinase | |
| *Mcm6* | -3.03 | | 1.82E-08 | | EC:3.6.4.12 | | minichromosome maintenance complex component 6 | |
| *Prim1* | -1.47 | | 1.50E-07 | | EC:2.7.7.102 | | DNA primase, p49 subunit | |
| *Cdk1* | -1.63 | | 1.14E-05 | | EC:2.7.11.22 | | cyclin-dependent kinase 1 | |
| *Espl1* | -1.36 | | 2.20E-05 | | EC:3.4.22.49 | | extra spindle pole bodies 1, separase | |
| *Mad2l1* | -1.13 | | 3.29E-05 | | - | | MAD2 mitotic arrest deficient-like 1 | |
| *Mcm2* | -1.92 | | 1.51E-04 | | EC:3.6.4.12 | | minichromosome maintenance complex component 2 | |
| *Mcm3* | -1.49 | | 1.67E-04 | | EC:3.6.4.12 | | minichromosome maintenance complex component 3 | |
| *Cdc6* | -2.89 | | 1.68E-04 | | - | | cell division cycle 6 | |
| *Pole* | -1.48 | | 1.82E-04 | | EC:2.7.7.7 | | polymerase (DNA directed), epsilon | |
| *Cdc7* | -1.92 | | 2.60 E-04 | | EC:2.7.11.1 | | cell division cycle 7 | |
| *Cdc25c* | -2.00 | | 2.62E-04 | | EC:3.1.3.48 | | cell division cycle 25C | |
| *Lig1* | -1.66 | | 2.70E-04 | | EC.6.5.1.1 | | ligase I, DNA, ATP-dependent | |
| *Mcm4* | -1.41 | | 4.29E-04 | | EC:3.6.4.12 | | minichromosome maintenance complex component 4 | |
| *Mcm5* | -2.19 | | 4.72E-04 | | EC:3.6.4.12 | | minichromosome maintenance complex component 5 | |
| *Ccne2* | -1.03 | | 5.98E-04 | | - | | cyclin E2 | |
| *Cdkn1a* | -1.16 | | 6.56E-04 | | - | | cyclin-dependent kinase inhibitor 1A (P21) | |
| *Rfc4* | -1.01 | | 3.56E-03 | | - | | replication factor C (activator 1) 4 | |
| *Cdc45* | -1.32 | | 3.84E-03 | | - | | cell division cycle 45 | |
| *Orc1* | -2.31 | | 7.62E-03 | | - | | origin recognition complex, subunit 1 | |
| *Cdkn2b* | -1.38 | | 3.24E-02 | | - | | cyclin dependent kinase inhibitor 2B | |

**Table S5.** Mitochondria increased gene transcription of metabolic enzymes and protein

| **gene name** | **log2foldchange** | **p-value** | | **E.C.** | | **description** | |
| --- | --- | --- | --- | --- | --- | --- | --- |
| *Ugt3a1* | 1.93 | 2.61E-22 | EC 2.4.1.17 | | UDP glycosyltransferases 3 family, polypeptide A1 | |  |
| *Cyp2e1* | 1.78 | 2.87E-16 | EC:1.14.14.1 | | cytochrome P450, family 2, subfamily e, polypeptide1 | |  |
| *Nat8* | 2.13 | 1.26E-14 | EC 2.3.1.5 | | N-acetyltransferase 8 | |  |
| *Cyp4a12a* | 2.04 | 2.80E-07 | EC:1.14.14.80 | | cytochrome P450, family 4, subfamily a, polypeptide12a | |  |
| *Cyp2c54* | 1.39 | 5.15E-06 | EC:1.14.14.11 | | cytochrome P450, family 2, subfamily c, polypeptide54 | |  |
| *Gst1* | 0.70 | 5.23 E-05 | EC:2.5.1.18 | | glutathione S-transferase 1 | |  |
| *Sult1b1* | 0.73 | 3.94E-04 | EC:2.8.2.1 | | sulfotransferase family 1B, member 1 | |  |
| *Ugt2b1* | 1.20 | 3.22E-03 | EC:2.4.1.17 | | UDP glucuronosyltransferase 2 family, polypeptideB1 | |  |
| *Ugt2a3* | 0.50 | 4.41E-03 | EC:2.4.1.17 | | UDP glucuronosyltransferase 2 family, polypeptideA3 | |  |
| *Cyp4a32* | 1.18 | 3.21E-02 | EC:2.4.1.17 | | cytochrome P450, family 4, subfamily a, polypeptide32 | |  |
| *Ugt2b37* | 1.16 | 3.71E-02 | EC:2.4.1.17 | | UDP glucuronosyltransferase 2 family, polypeptideB37 | |  |
| *Marc2* | 0.41 | 2.07E-02 | - | | mitochondrial amidoxime reducing component 2 | |  |
| *Gstz1* | 0.39 | 2.53E-02 | EC:5.2.1.2 | | glutathione transferase zeta 1 | |  |

**Table S6.** Mitochondria up-regulated gene transcriptions of a series of MUPs.

| **gene name** | **log2foldchange** | **p-value** | **description** |
| --- | --- | --- | --- |
| *MUP18* | 5.03 | 1.89E-05 | major urinary protein 18 |
| *Mup3* | 2.03 | 3.25E-05 | major urinary protein 3 |
| *Mup10* | 5.60 | 9.26E-05 | major urinary protein 10 |
| *Mup2* | 3.66 | 2.24E-04 | major urinary protein 2 |
| *Mup22* | 5.62 | 2.31E-04 | major urinary protein 22 |
| *Mup11* | 5.38 | 2.78E-04 | major urinary protein 11 |
| *Mup7* | 7.92 | 4.28E-04 | major urinary protein 7 |
| *Mup13* | 5.19 | 5.13E-04 | major urinary protein 13 |
| *Mup16* | 3.64 | 6.39E-04 | major urinary protein 16 |
| *Mup19* | 5.58 | 2.84E-03 | major urinary protein 19 |
| *Mup12* | 4.56 | 3.54E-03 | major urinary protein 12 |
| *Mup14* | 3.76 | 4.27E-03 | major urinary protein 14 |
| *Mup1* | 4.48 | 1.17E-02 | major urinary protein 1 |
| *Mup17* | 4.25 | 1.78E-02 | major urinary protein 17 |
| *Mup15* | 4.88 | 2.35E-02 | major urinary protein 15 |
| *Mup21* | 1.18 | 2.76E-02 | major urinary protein 21 |
| *Mup8* | 2.96 | 4.97E-02 | major urinary protein 8 |

**Table S7.** Mitochondria up-regulated gene transcriptions of UPR^mt^ markers.

| **gene name** | **log2foldchange** | **p-value** | | **EC** | | **description** | | |
| --- | --- | --- | --- | --- | --- | --- | --- | --- |
| *Dnaja4* | 0.67 | | 2.97E-03 | | - | | DnaJ heat shock protein family (Hsp40) |  |
| *Hsp70* | 1.04 | | 2.67E-02 | | - | | heat shock protein 70 |  |
| *Hsp60* | 0.51 | | 2.82E-02 | | - | | heat shock protein 60 |  |
| *AFG3L2* | 0.16 | | 3.86E-02 | | EC:3.4.24.2 | | AFG3-like AAA ATPase 2 |  |
| *lonp1* | 0.21 | | 4.22E-02 | | EC:3.4.21.53 | | lon peptidase 1, mitochondrial |  |
| *Hsph1* | 0.45 | | 4.39E-02 | | - | | heat shock 105kDa/110kDa protein 1 |  |
| *Clpp* | 0.40 | | 4.44E-02 | | EC:3.4.21.92 | | caseinolytic mitochondrial matrix peptidaseproteolytic subunit |  |
| *atf5* | 0.31 | | 4.82E-02 | | - | | activating transcription factor 5 |  |
